# Naso-Pharyngeal Growths

**Published:** 1909-05-01

**Authors:** 


### Laryngology and Rhinology.

### NASO-PHARYNGEAL GROWTHS.

True tumours, under which heading adenoid vegetations are not included, are somewhat uncommon in the region of the naso-pharynx, but they nevertheless occur with more frequency than is generally realised; for they undoubtedly often escape recognition unless a posterior rhinoscopic examination is made whenever there are symptoms of nasal obstruction or catarrh. Several varieties of "naso-pharyngeal polypus" are to be distinguished.

In cases of ordinary nasal *mucous polypus* it is by no means uncommon to find masses of these growths extending into, or even completely filling, the naso-pharynx; sometimes the formation of these polypi is confined to the regions of the posterior ethmoidal cells and sphenoidal sinuses, and little or nothing of them can be seen by examination of the anterior nares. They do not, however, arise in the naso-pharynx, but always have their attachments within the nose, and protrude backwards into the naso-pharyngeal cavity. Their causation, pathology, and treatment are the same as that of the ordinary nasal polypi. They are often associated with disease of the posterior accessory sinuses, are extremely difficult to snare, and are generally best treated by thorough curettage under a general anæsthetic. Hence the naso-pharynx should be examined with the rhinoscopic mirror in all cases of nasal polypi before removal with the snare; for, if it is afterwards found necessary to give a general anæsthetic to remove the masses in the naso-pharynx, the patient will not be grateful for the discomfort already inflicted. With large masses of polypi in the naso-pharynx are better removed, both anterior and posterior, all at one sitting under anæsthesia.

Another form of mucous polypus in the naso-pharynx is of interest, though seldom dwelt upon in text-books. It is indistinguishable in appearance from the usual nasal polypus, but occurs without any association with polypi within the nares. It is a single unilateral growth which hangs backwards into the naso-pharynx from a long pedicle attached within the nares. It is found in young people between 15 and 25, and far more commonly in the female sex. It is said by Killian, and his opinion is supported, that the pedicle is attached to the mucous membrane of the maxillary antrum just within the ostium, and that it passes into the nares through this or through the accessory ostium, which frequently exists further back above the middle turbinal body. It should be removed by traction so as to draw away the pedicle, and this is easily done by means of polypus forceps guided by the finger in the naso-pharynx. There is no marked tendency to recurrence. There is generally no sign of antral disease, and certainly no antral suppuration, in these cases. These single polypi occasionally reach a very large size and completely fill and, as it were, form a cast of, the nares and naso-pharynx.

*Fibromata*, which are those generally described in the surgical text-books under the name of "naso-pharyngeal polypus," are far more formidable. They, also, are almost invariably found between the ages of 15 and 25, but nearly exclusively in males. They do not merely project into the naso-pharynx, but grow from its roof. They are not truly malignant, for they do not produce metastases or infect glands, but they show a marked tendency to recur locally. They are vascular, bleed readily, and often profusely. They are composed of fibrous tissue, varying from well-formed fibres to young spindle-cells which imitate the appearance of sarcoma; the more the tumour aproaches the spindle-cell type, the more rapid is its growth and the greater the tendency to hæmorrhage and to recurrence after removal. This latter also depends very largely on the breadth of its attachment, which may be sessile or by a fine pedicle. The method of removal must also depend on the attachment, which should be carefully examined with the finger before any operation is undertaken. When the pedicle is quite thin, the growth may be easily removed with forceps or cold snare; but if it be thicker severe bleeding must be anticipated and the galvano-cautery snare should be employed. When there is a broad attachment removal is very difficult, and recurrence is probable; it is usually best, after preliminary laryngotomy, to split the soft palate and cut away the hard palate until the base of the tumour is thoroughly exposed from the mouth and to remove it by chiselling away the bone to which it is attached, finally uniting the divided soft tissues of the palate.

Among *Malignant Growths* sarcoma is extremely rare, unless the more sessile and rapidly growing fibromata be considered as such. Epithelioma, however, is not very uncommon, though often undetected until it is far advanced and involves the cervical glands. In every case of apparently malignant glands without obvious cause the naso-pharynx should always be examined for the primary growth. As this part is not greatly concerned in deglutition, pain is absent or very slight until the later stages. The first complaint is often deafness, owing to obstruction of the eustachian tubes. Rhinoscopic examination discloses an irregular lobulated mass, and the characteristic hardness of the growth can be made out on palpation. These growths frequently remain undiscovered until the secondary glandular involvement has progressed so far as to contraindicate operation; but if they are diagnosed before this has occurred removal may be attempted with some prospect of success. The operation may be performed through the mouth after splitting the palate, as described above for the removal of fibromata, and this method gives a remarkably good view of the part; but if the growth has penetrated into the nasal cavity it can only be thoroughly extirpated after partial excision of the upper jaw. In some cases an osteoplastic resection of the jaw may be recommended, the bone being restored to its position at the end of the operation. A preliminary laryngotomy should always be performed, and the pharynx plugged to prevent the entrance of blood into the lungs.

